# Picocyanobacteria containing a novel pigment gene cluster dominate the brackish water Baltic Sea

**DOI:** 10.1038/ismej.2014.35

**Published:** 2014-03-13

**Authors:** John Larsson, Narin Celepli, Karolina Ininbergs, Christopher L Dupont, Shibu Yooseph, Bigitta Bergman, Martin Ekman

**Affiliations:** 1 Department of Ecology, Environment and Plant Sciences, Stockholm University, Science for Life Laboratory, Solna, Sweden; 2 Microbial and Environmental Genomics, J Craig Venter Institute, San Diego, CA, USA; 3 Informatics Group, J Craig Venter Institute, San Diego, CA, USA

**Keywords:** cyanobacteria, phycobilisome, pigment, horizontal gene transfer, Baltic Sea, ecology

## Abstract

Photoautotrophic picocyanobacteria harvest light via phycobilisomes (PBS) consisting of the pigments phycocyanin (PC) and phycoerythrin (PE), encoded by genes in conserved gene clusters. The presence and arrangement of these gene clusters give picocyanobacteria characteristic light absorption properties and allow the colonization of specific ecological niches. To date, a full understanding of the evolution and distribution of the PBS gene cluster in picocyanobacteria has been hampered by the scarcity of genome sequences from fresh- and brackish water-adapted strains. To remediate this, we analysed genomes assembled from metagenomic samples collected along a natural salinity gradient, and over the course of a growth season, in the Baltic Sea. We found that while PBS gene clusters in picocyanobacteria sampled in marine habitats were highly similar to known references, brackish-adapted genotypes harboured a novel type not seen in previously sequenced genomes. Phylogenetic analyses showed that the novel gene cluster belonged to a clade of uncultivated picocyanobacteria that dominate the brackish Baltic Sea throughout the summer season, but are uncommon in other examined aquatic ecosystems. Further, our data suggest that the PE genes were lost in the ancestor of PC-containing coastal picocyanobacteria and that multiple horizontal gene transfer events have re-introduced PE genes into brackish-adapted strains, including the novel clade discovered here.

## Introduction

Unicellular picocyanobacteria are widespread in aquatic ecosystems across the globe (Scanlan *et al.*, 2009). Being photoautotrophic and often present at densities of over one million cells per ml seawater, they contribute significantly to global carbon cycling (Olson *et al.*, 1990; Jardillier *et al.*, 2010). Picocyanobacteria, which are defined as being <2 μm in size, belonging to the *Synechococcus/Cyanobium* group form a well-defined clade within the cyanobacterial lineage termed ‘cluster 5' (Urbach *et al.*, 1998; Fuller *et al.*, 2003). This cluster is further divided into subcluster 5.1, which mostly contains strictly marine strains, subcluster 5.2 containing strains from a range of salinities and the least studied 5.3 subcluster that contains only a few recognized strains. In this article, we use the term ‘picocyanobacteria' to denote *Synechococcus* and *Cyanobium* strains belonging to cluster 5.

Picocyanobacteria exhibit a range of colours depending on the composition of multi-subunit protein structures termed phycobilisomes (PBS), which complement light absorption via chlorophyll. PBS composed mainly of the pigment phycocyanin (PC) absorb red light, giving cells a blue-green appearance, whereas phycoerythrin (PE) absorbs green light and give the cells a red colour. The PBS genes are found in conserved genomic regions in cluster 5 picocyanobacteria and the organization of these regions correlates with one of three pigmentation types (1–3) (Six *et al.*, 2007). To avoid confusion with the subcluster phylogenetic notation for picocyanobacteria, we designate these types with roman numerals (I, II and III). Table 1 shows characteristic properties and gene content for the different pigment types. Type I gene clusters contain only the PC subunit genes *cpcBA* and are found in blue-green strains adapted to turbid coastal brackish and limnic waters where red light dominates. Types II and III gene clusters in addition contain the phycoerythrin-I (PEI) subunit genes *cpeBA* and are found in red strains adapted to clear waters of the open ocean where blue wavelengths dominate. Type III strains also contain the *mpeBA* genes encoding phycoerythrin-II (PEII) and are further divided into subtypes a, b and c depending on the ratio of the chromophores phycoerythrobilin and phycourobilin bound to the PBS (Six *et al.*, 2007). Finally, certain type III strains, belonging to subtype d, vary their phycourobilin: phycoerythrobilin ratio depending on light conditions, a mechanism known as type 4 chromatic acclimation (CA4) (Palenik, 2001; Gutu and Kehoe, 2012).

Genomes of 13 marine picocyanobacterial strains with types II or III pigmentation have been sequenced to date (Table 2), and in nine of these the PBS gene cluster has been studied in detail (Six *et al.*, 2007). In contrast, analyses of pigment genes in non-marine strains (belonging to the 5.2 subcluster) have largely been limited to PCR-based analyses of PC and/or PE genes (Ivanikova *et al.*, 2007; Haverkamp *et al.*, 2008, 2009; Somogyi *et al.*, 2010; Felföldi *et al.*, 2011; Jasser *et al.*, 2011). Only five genome sequences are available for the 5.2 subcluster (Table 2) and among these the PBS gene cluster has only been fully characterised in the type I strain WH5701 (Six *et al.*, 2007). This genome sequencing bias has made it difficult to draw conclusions on the evolution, including the impact of horizontal gene transfer (HGT), of the PBS gene cluster in picocyanobacteria, especially in the 5.2 subcluster.

**Figure 1:**
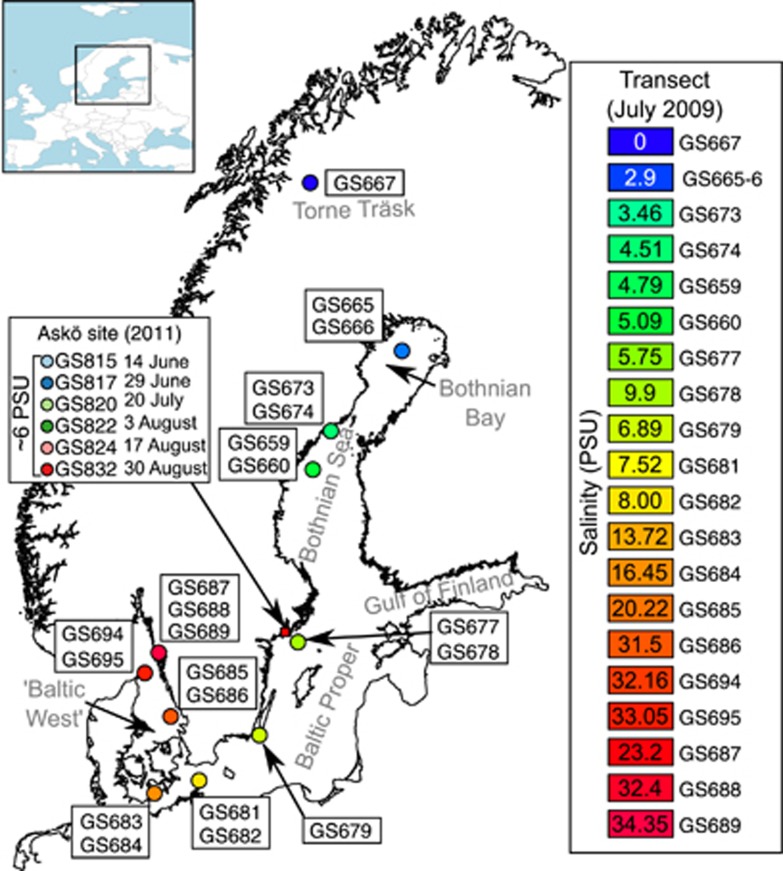
Map of the Baltic Sea area sampling sites. The map shows the location of sampling sites in the transect study conducted in 2009 (GS665–GS695). Samples are colour coded according to salinity (see legend on the right). The Askö site and collection time points for the summer of 2011 (GS815–GS832) are marked on the map and in the left legend. The inset map of Europe shows the location and extent of the Baltic Sea.

In order to expand our knowledge of the biogeography of PBS pigment genotypes and the organization and evolution of PBS gene clusters in cluster 5 picocyanobacteria, we analysed genomes of uncultivated picocyanobacterial populations in a total of 94 aquatic samples separated spatially along a 1800 km salinity gradient (Dupont *et al.*, 2014) and temporally across a summer growth season in the Baltic Sea. The Baltic Sea is a temperate brackish water body, with a stable north to south salinity gradient (2–8 practical salinity units (PSU)), connected to fully marine (via the North Sea) and limnic (via inland lakes and rivers) waters (Figure 1). Our data show large variations in pigment gene types between different salinity habitats with small seasonal variations in the brackish community. Notably, we identified a novel type of the PBS gene cluster in the dominant subcluster 5.2 picocyanobacteria. These results further our understanding of molecular adaptations to light in brackish and coastal water ecosystems and the evolution of light-harvesting genes in cyanobacteria.

## Materials and methods

### Sampling and sequencing

Our data set comprised 21 samples collected along a 1800 km long transect in the Baltic Sea in July 2009 and 6 samples collected from near-shore waters in the central Baltic at six time points during a summer season in June to August in 2011 (Figure 1). The 2009 transect spans the northern Bothnian Bay to the southern Baltic Proper as well as marine waters in the Danish straits, Kattegat and Skagerrak here referred to collectively as ‘Baltic West' (Figure 1). One sample was also taken in the freshwater lake Torne Träsk in the north of Sweden. Several depths were sampled at all but two locations (GS667 and GS679) in the 2009 transect, while the 2011 samples were taken from surface waters. Two samples in the 2009 transect were taken at >70 m depth (GS678 and GS689) and were not included in analyses because of the low number of cyanobacterial reads. All samples were size fractionated into 0.1–0.8, 0.8–3.0 and 3.0–200 μm size classes. In addition, viral fractions were collected for seven samples in the 2009 transect and for all samples in the 2011 time series by concentration of the <0.1 μm filtrate using tangential flow filtration as described previously (Williamson *et al.*, 2012). Sample details and environmental data are given in Supplementary Table S1. The 2009 samples were shotgun sequenced using 454 pyrosequencing. The 2011 samples were sequenced using paired end Illumina MiSeq and HiSeq with 150-bp and 100-bp insert sizes, respectively.

### Identification of PBS subunits

PC (*cpcBA*), PEI (*cpeBA*) and PEII (*mpeBA*) subunits were identified in the Baltic Sea metagenomic data sets using reciprocal best blast searches. In a first step, protein sequences from sequenced picocyanobacterial genomes in each subunit category were used to query all metagenomic proteins. Retained (*E*-value <1e^−10^) metagenomic sequences were then searched against the NCBI RefSeq database and queries with hits to either of the original phycobilisome proteins were kept. As a result of thorough annotations of the phycobilisome gene cluster (Six *et al.*, 2007) our reciprocal homology search allowed us to efficiently detect relevant genes, although it should be noted that the biochemical function has not been shown for the genes we identified in this study.

### Metagenomic assembly

An assembly from the 2009 transect data set was generated using 698 865 454 reads classified as cyanobacterial using Newbler (Margulies *et al.*, 2005) at 98% identity threshold via the 454 meta-assembler hosted at CAMERA (Seshadri *et al.*, 2007). For the 2011 time series study, each sample (filter per collection date) was assembled using Ray Meta (Boisvert *et al.*, 2012) with a 31 or 41 kmer size. Assembly statistics are given in Supplementary Table S2.

### Phylogenetic analyses

For all phylogenetic analyses, amino-acid sequence alignments were constructed using Muscle v. 3.8.31 (Edgar, 2004). Amino-acid substitution models were selected based on the Akaike information criterion using ProtTest v. 3.0 (Darriba *et al.*, 2011) and maximum-likelihood (ML) phylogenies were constructed using RAxML v. 7.3.5 (Stamatakis, 2006) with a gamma model of rate heterogeneity estimated by the program and a partitioned model setup for concatenated alignments (when applicable). A total of 1000 bootstrap replicates were conducted for each analysis unless otherwise specified. Trees were visualized in interactive tree of life (Letunic and Bork, 2007).

#### Core-genome

Evolutionary conserved genes were identified in the metagenomic data set and in 126 cyanobacterial genomes using the AMPHORA2 pipeline (Wu and Scott, 2012). A core-genome ML phylogeny was constructed from a concatenated alignment of translated amino-acid sequences for six ribosomal genes (*rplEFNP* and *rpsEM*) found in all of the 126 cyanobacterial reference genomes and on 11 contigs from samples collected throughout the 2011 summer season.

#### PBS subunits

PBS subunits in picocyanobacterial reference genomes and on assembled contigs that contained both the alpha and beta subunits in each category were used to construct phylogenetic reference trees. Amino-acid sequences for the subunits were aligned individually with Muscle and the alignments curated manually in Jalview (Waterhouse *et al.*, 2009) before concatenating.

#### Evolutionary placement of reads

The evolutionary placement algorithm in RAxML (Berger *et al.*, 2011) was used to insert sequence fragments into corresponding (core-genome or PBS subunit) reference trees.

Detailed experimental procedures are given in the Supplementary Information. All sequence and annotation data for the 2009 transect are available at CAMERA under accession number CAM_P_0001109. Assembled contigs and additional gene sequences from the 2011 time series are available in the Supplementary Information.

## Results

The majority of sequences identified in this study originated from the 0.8 to 3.0 μm size fraction (72%) and to a lesser extent from the 0.1 to 0.8 and 3.0 to 200 μm size fractions (5% and 23%, respectively). Therefore, we designate the environmental sequences described here as belonging to ‘picocyanobacteria' (<2 μm) although our methodology does not allow us to discriminate between cells with sizes in the 0.8–3.0 μm range.

### Picocyanobacterial phylogeny and distribution

**Figure 2:**
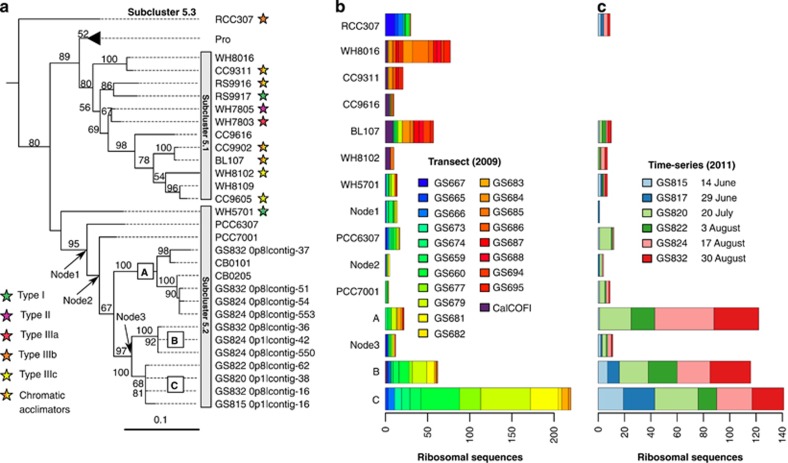
Picocyanobacterial phylogeny and distribution. (**a**) ML phylogeny of six ribosomal proteins found in 126 cyanobacterial genomes and 11 contigs in the 2011 time series assembly. Contig names include the sample id (GS815–GS832), filter fraction (0p1=0.1–0.8 μm, 0p8=0.8–3.0 μm and 3p0=3.0–200 μm) and contig number. Bootstrap support >50% (1000 replicates) are shown at nodes and the scale bar indicates number of expected substitutions per site. The tree was rooted using *Gloeobacter violaceus* PCC7421 and only the *Synechococcus* cluster 5 subtree is shown (see the Supplementary material for the full phylogeny). The *Prochlorococcus* clade has been collapsed. Arrows indicate nodes introduced after phylogenetic placement of sequence reads. The coloured stars indicate pigment type for reference strains (only shown for those studied in Six *et al.*, 2007). See Table 2 for genome abbreviations. (**b**, **c**) Distribution of reads from the 2009 transect (**b**) and 2011 time series (**c**) in the phylogeny in **a**. CalCOFI, combined reads from metagenomic samples off the west coast of California (Zeigler Allen *et al.*, 2012).

We constructed a ML phylogeny based on a concatenated alignment of six ribosomal proteins in 126 cyanobacterial reference genomes, and 11 contigs from the 2011 time series assembly (Figure 2a). As we focused on PBS genes in cluster 5 picocyanobacteria, the phylogeny of this clade is shown in Figure 2a, while the full cyanobacterial phylogeny is presented in the Supplementary Material (Supplementary data set 1). Ribosomal sequence fragments obtained from the two metagenomic data sets (transect and time series) were inserted into the phylogeny, while keeping the original tree intact, to determine the distribution (in space and time) of picocyanobacterial phylotypes in the Baltic Sea. Contigs in the time series data set all grouped within subcluster 5.2 and partitioned between a clade formed by the reference strains CB0101 and CB0205 (clade A) and a clade composed of two subclades B and C without reference strains (Figure 2a). Environmental sequences belonging to the two latter subclades, and in particular subclade C, dominated all low-salinity and brackish sites in the Baltic Sea transect as well as the entire season in 2011 (Figures 2b and c). Members of clade A made up a minor part of the picocyanobacterial population in the Baltic Sea transect in July 2009 (Figure 2b) but became more abundant later in the summer in 2011 (Figure 2c). In contrast, subcluster 5.1 picocyanobacteria were predominantly found in samples from the marine Baltic West (13–35 PSU) and were infrequent in the time series data set (∼6 PSU). A majority of these marine genotypes clustered in subclades containing strains capable of CA4 (Palenik, 2001) such as BL107 and CC9311 or their close relatives (for example, WH8016; Figure 2b). Sequences closely related to the subcluster 5.3 strain RCC307 (Figure 2a), although in low abundance, were found at freshwater and low-salinity sites (0–3 PSU; Figure 2b) as well as in early and late summer samples of 2011 (Figure 2c).

### Spatial distribution of PBS subunit genes in the Baltic Sea transect

**Figure 3:**
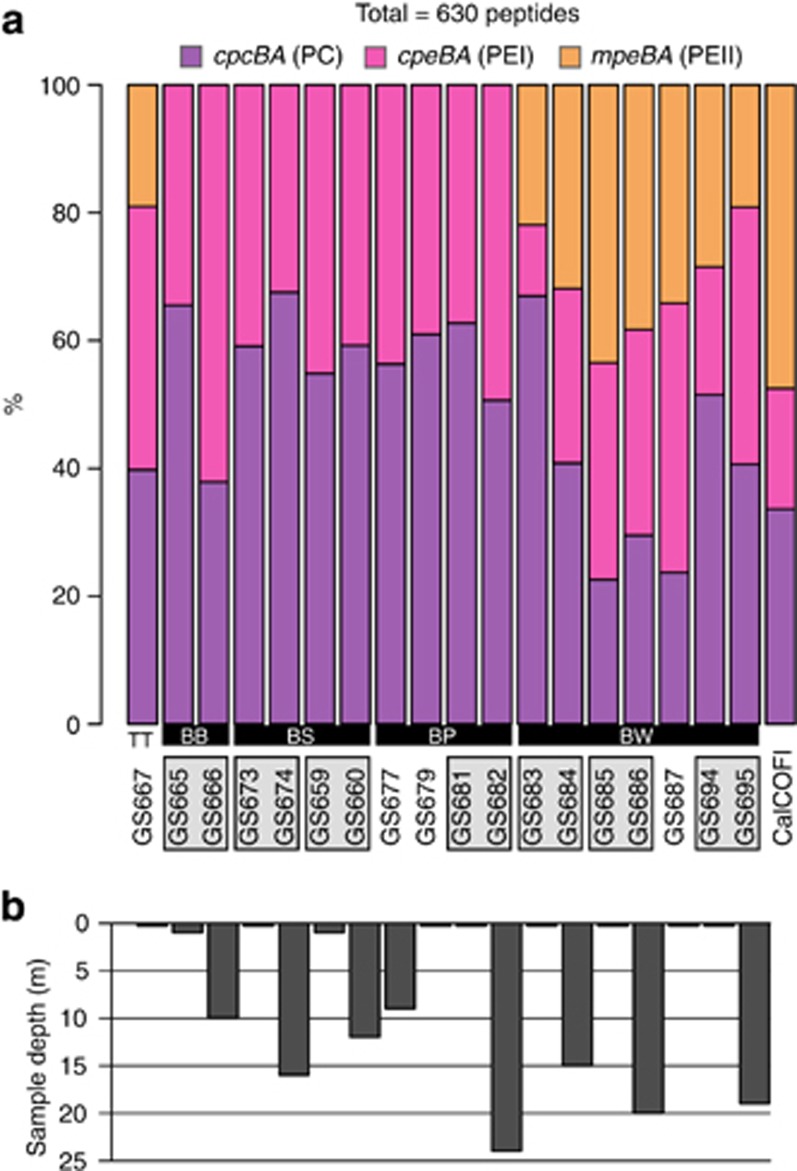
Proportions of PBS genes. (**a**) Distribution of PC (c*pc*BA), PEI (c*pe*BA) and PEII (*mpeBA*) subunit genes in the metagenomic data set. The number of genes was normalized against the picocyanobacterial RecA protein and the graph shows the (normalized) relative proportion of each PBS subunit type at each site in the Baltic Sea metagenomic transect. The subbasin/location for samples is indicated by the abbreviations in the lower margin: BB, Bothnian Bay; BS, Bothnian Sea; BP, Baltic Proper; BW, Baltic West; C=CalCOFI (west coast of California); TT, Lake Torne Träsk. (**b**) Sampling depth at each site.

PC and PEI subunits showed a relatively even distribution in the freshwater/brackish Baltic Sea samples (GS665–GS682; Figure 3) with no significant differences between samples collected at the surface and deeper (max 24 m) within the water column. However, PEII subunits, which were frequent in the marine samples (GS683–GS695) and were also present in lake Torne Träsk (GS667) were not found in the brackish water sites (GS665–GS682; Figure 3). The combined average proportion of PBS subunit sequences in the marine CalCOFI metagenome (off the west coast of California; Zeigler Allen *et al.*, 2012) was similar to the marine west coast samples in the Baltic Sea area (Figure 3).

### Phylogeny of phycobiliprotein genes from the Baltic Sea

**Figure 4:**
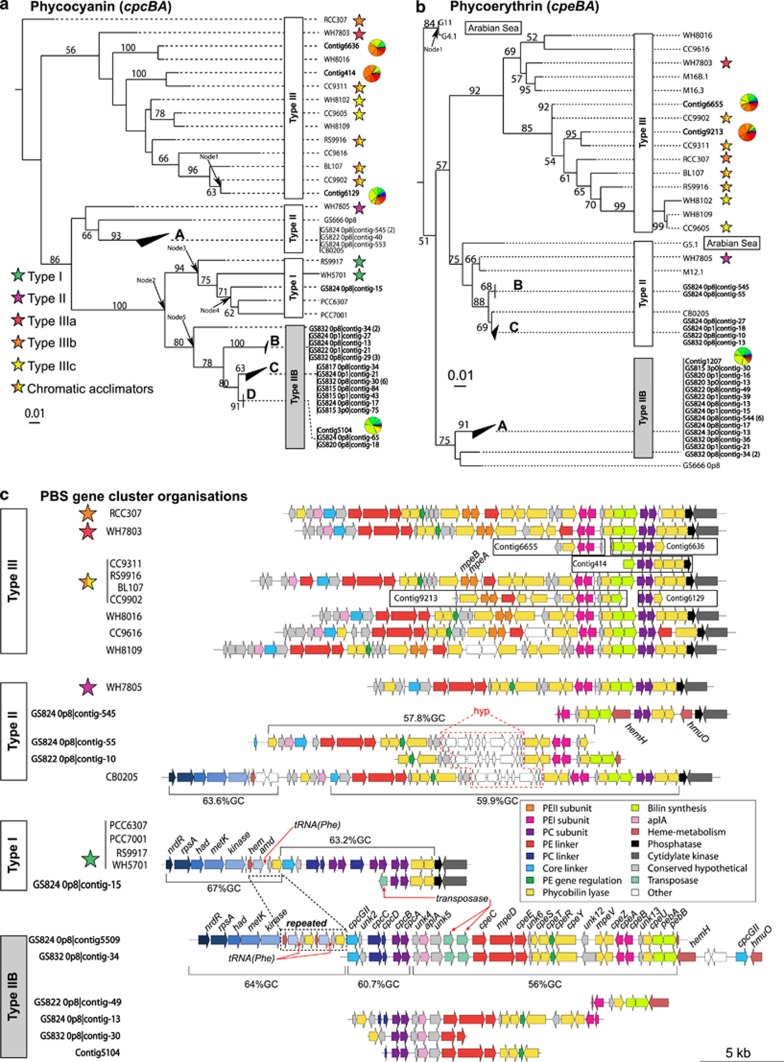
Phylogeny of phycobiliproteins and structure of the PBS operons. ML phylogenies of concatenated amino-acid alignments of PC (*cpcBA*) (**a**) and PE (c*peBA*) (**b**) subunits from reference genomes and assembled contigs from the Baltic Sea metagenomes. The trees were rooted with *Gloeobacter violaceus* (data not shown). Bootstrap (1000 replicates) support >50% is shown at nodes and the scale bar indicates number of expected substitutions per site. Stars as in Figure 2. For contigs in the 2009 global assembly, pie charts show the read distribution from each site. Numbers in parenthesis next to contigs indicate the number of contigs with identical PC or PE subunits. Arrows show internal nodes introduced by phylogenetic placement of sequence fragments (see Figure 5). (**c**) PBS operon structures of picocyanobacteria in the data set. Operon schematics are shown for each pigment type and for assembled contigs in the Baltic Sea data set. For contigs that occupy the same position in the PC and PE phylogenies, only the longest contig is shown. The region designated 'hyp' in type II genomes indicates a 4.4-kb stretch of conserved hypothetical genes. *amd*, predicted amidophosphoribosyltransferase; *had*, haloacid dehalogenase-like hydrolase; *hem*, putative heme iron utilization protein; *kinase*, carbohydrate kinase, FGGY family protein; *metK*, *S*-adenosylmethionine synthetase; *nrdR*, transcriptional regulator NrdR; *rpsA*, 30S ribosomal protein S1.

The phylogeny obtained from concatenated PC subunits showed that the overwhelming majority of PC subunits in our data set did not cluster with those of previously sequenced isolates, instead forming a well-supported sister-clade to those from type I strains (Figure 4a). Of the 23 contigs that clustered within this novel clade, 18 were sufficiently long and allowed us to identify PEI-associated genes downstream of the *cpcBA* operon (see Supplementary data set 2). The remaining five contigs were shorter than 2 kbp and did not contain enough sequence information flanking the *cpcBA* operon. However, an analysis of GC content for all *cpcBA* operon sequences (including the intergenic sequence between the beta and alpha subunits) in the PC phylogeny showed that the %GC of the novel clade was significantly lower than in both the type II and type I clades (Supplementary Figure S1). On the basis of these results, we designate the novel clade ‘type IIB'. The type IIB clade was further divided into multiple subclades (B–D, Figure 4a) with high-to-moderate bootstrap support. Sequences within these subclades were highly conserved, showing ∼99% identity for both amino-acid and nucleotide alignments. One contig from the 2009 transect assembly that contained reads mostly from the low-salinity and brackish samples clustered within one of these type IIB subclades (Contig5104; Figure 4a). In contrast, contigs from the same assembly that were dominated by reads from marine samples clustered within the type III clade, as neighbours to 5.1 subcluster strains WH8016, CC9311 and CC9902, thus consistent with the ribosomal phylogeny (Figure 2). A few contigs from the Askö 2011 time series grouped within the type II clade and were closely related to the subcluster 5.2 strain CB0205, while only one contig clustered with type I genomes.

The position of Baltic Sea picocyanobacteria in the PE phylogeny (Figure 4b) was largely congruent with the PC tree. Type IIB genotypes were again well separated from both the type II and type III clades, as well as from the G11/G4.1 and G5.1 *Cyanobium* strains from the Arabian Sea (AS) (Everroad and Wood, 2006). Analysis of the RNA polymerase subunit RpoC1 further confirmed that Baltic Sea picocyanobacteria were not closely related to the AS *Cyanobium* lineage (Supplementary Figure S2). Contigs with both PC and PEI subunits grouped within the same clades in both trees with only minor subclade deviations. In addition, one PEI-containing contig, Contig1207, from the 2009 transect assembly clustered within the type IIB clade and showed a read distribution highly similar to Contig5104 in the PC tree. Contigs assembled primarily from reads in the marine samples again grouped with subcluster 5.1 strains CC9902 and CC9311. The type IIB PEI clade could not be resolved into subclades, indicating a high degree of PEI subunit similarity. The branching point of the type IIB clade occurred deeper in the PEI tree, compared with the PC phylogeny, with the G11/G4.1 strains clustering at the base of the tree. Although deep branching nodes in the phylogeny had relatively low bootstrap support the overall topology was highly similar to that found previously (Everroad and Wood, 2012). Approximately unbiased tests of alternative PEI topologies rejected branching patterns with the type II at the base of the tree with the AS strains sister to the type III and type IIB clades, as well as a topology with the type III and type IIB clades sister to the type II and AS clades (Supplementary Figure S3).

### Novel PBS gene cluster arrangement in Baltic Sea picocyanobacteria

The genomes of uncultivated Baltic Sea picocyanobacteria exhibited several unique genetic features. The PC subunit genes *cpcBA* and *cpcGII*, as well as *cpcCD* linkers on type IIB contigs were located upstream of the PEI-associated genes, at the opposite end of the PBS gene cluster, thereby contrasting all other reference genomes and Baltic Sea contigs with PEI genes (Figure 4c). Importantly, the *cpcCD* linker genes were not found in any other PEI-containing genome (Figure 4c and (Six *et al.*, 2007)). The two longest type IIB contigs, GS832_0p8|contig-34 and GS824_0p8|contig-5509, contained identical PC and PEI subunit protein sequences and showed 99% (21057/21066) overlapping nucleotide identity. The region upstream of *cpcGII* on the latter contig was highly similar to the same region in subcluster 5.2 genomes, but contained a 3977-bp repeated segment that included the phenylalanine tRNA gene. A ML phylogeny of the 30S ribosomal protein S1 coded for by *rpsA* (Figure 4c) located further upstream on contig-5509 positioned this contig within the 5.2 subcluster, as a sister-group to the CB0205/0101 strains (Supplementary Figure S4A). Interestingly, contig-34 harboured two copies of the PBS core linker *cpcGII*, situated at either end of the gene cluster. The products of the two *cpcGII* showed 53% AAID and exhibited different phylogenies (Supplementary Figure S4B). Although the CpcGII protein encoded by the gene at the start of the gene cluster clustered with CpcGII from WH5701 and CB0101, the second CpcGII protein grouped with the *Cyanobium* strains PCC7001 and PCC6307. We also found two transposase genes situated between the conserved hypothetical gene *unk5* and the PEI linker gene *cpeC* on contig-34 and contig-5509. No transposases were identified in any of the other type IIB gene clusters, but one contig within the type I PC clade contained a transposase gene upstream of *cpcBA* (Figure 4c).

A common feature of types II and IIB Baltic Sea picocyanobacteria was the position of the ferrochelatase gene *hemH* and the heme oxygenase gene *hmuO* within the PBS gene cluster (Figure 4c), two genes that are situated well apart from the phycobilisome genes in all other picocyanobacterial genomes. In types II and IIB strains, *hemH* was found directly downstream of the bilin biosynthesis genes *pebAB*. The *hmuO* gene was located downstream of the *cpcEF* lyase genes in type II strains and downstream of the second *cpcGII* gene copy on the type IIB contig-34, which did not contain *cpcEF*. Phylogenetic analysis of the HemH protein showed that all type II contigs formed a well-supported clade, while the type IIB contigs 34 and 5509 were separated from other type IIB genotypes belonging to PEI clade A (Supplementary Figure S4C). The HmuO phylogeny, on the other hand, clustered the type II contig-545 and type IIB contig-34 (Supplementary Figure S4D).

Interestingly, PBS gene clusters of type II Baltic Sea picocyanobacteria contained a stretch of conserved hypothetical genes between the phycobilin lyases *cpeY* and *mpeV* ('hyp' Figure 4c). This ∼4.4-kb genomic region showed 97% nucleotide identity to the same region in strain CB0205, but was not found in any other strain. Furthermore, the type II GS824_0p8|contig-553 clustered with strain CB0205 both in the core-genome phylogeny (Figure 2a) and the PC tree (Figure 4a). These results show that type II picocyanobacteria in the Baltic Sea are closely related to and share conserved PBS gene cluster features with the subcluster 5.2 strain CB0205.

The accuracy of these results was supported by the use of two assembly methods (Ray Meta and Newbler) that gave highly congruent results on two different sets of data (2011 time series and 2009 transect). In addition, we specifically observed the unique *cpcA*-*unk4* gene order seen in type IIB gene clusters on several raw 454 sequences obtained from brackish waters in the 2009 transect study (Supplementary Figure S5).

### Comparative analysis of pigment type distributions

As PC and PEI phylogenies group picocyanobacteria according to pigment phenotype and PBS gene cluster organization (Figures 4a and b; and Six *et al.*, 2007; Haverkamp *et al.*, 2008, 2009), we reasoned that the phylogenetic position of PC and PEI sequences reflects the type of PBS gene cluster in the corresponding picocyanobacterial genomes. We therefore placed PC and PEI sequence fragments from the 2009 Baltic Sea transect and 2011 time series data sets, as well as sequences from several studies that sampled picocyanobacterial pigment genes from various habitats across the globe (Supplementary Table S3), into each respective phycobiliprotein phylogeny.

**Figure 5:**
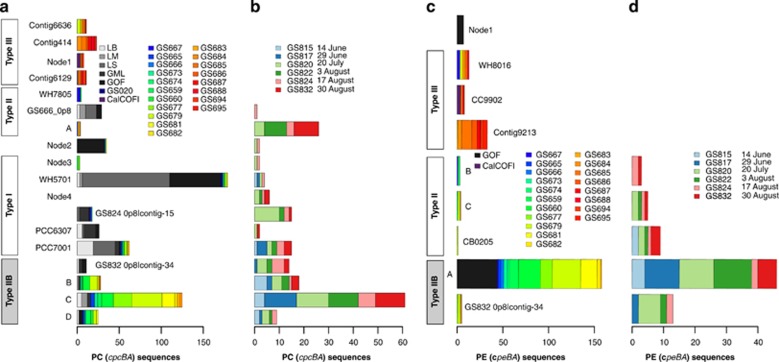
Distribution PBS operons inferred from phylogenetic placement of PC and PE subunits. Amino-acid sequences for PC (*cpcBA*) and PE (*cpeBA*) sequences were inserted into the respective phylogenetic tree (shown in Figures 4a and b) using the evolutionary placement algorithm in RAxML. Each bar plot shows the distribution of sequences from various samples in the corresponding phylogeny. If sequences were inserted directly on a terminal leaf, the strain/contig name is shown in the plot. For sequences inserted into internal nodes, the node name is given and its position in the tree is shown in Figure 4a or Figure 4b. For collapsed clades, the combined distribution of sequences in the clade is shown. (**a**, **b**) PC sequences from the 2009 transect and previous studies (**a**) and from the 2011 time series (**b**). (**c**, **d**) PE sequences from the 2009 transect and previous studies (**c**) and from the 2011 time series (**d**). Pigment type clade designations are shown in boxes with the novel type IIB clade shaded in grey. CalCOFI, West coast of California (California Current); GML, Great Mazurian Lakes; GOF, Gulf of Finland; GS020, Lake Gatun; LB, Lake Balaton; LM, Lake Mondsee.

Our data showed that Baltic Sea type IIB genotypes dominated the low-salinity and brackish sites in the 2009 transect (Figures 5a and c) as well as the entire 2011 summer season (Figures 5b and d). In contrast, environmental sequences from various freshwater habitats clustered almost exclusively with type I strains (WH5701, PCC7001 and PCC6307). However, a few sequences from habitats such as Lake Balaton in Hungary and Lake Mondsee in Austria as well as the Gulf of Finland (in the eastern Baltic Sea) grouped within the type IIB clade (Figure 5a) indicating that type IIB genotypes may make up a minor part of the population in these and other environments. Moreover, a number of PC sequences from picocyanobacteria in the Gulf of Finland (Haverkamp *et al.*, 2008, 2009) were inserted at the base of the type I and type IIB clades (see ‘Node2' in Figure 4a and sample ‘GOF' in Figure 5a). PEI sequences from the same study also clustered with the AS strains G11/G4.1 (Node1, Figures 4b and 5c). Type II genotypes were essentially missing from most sites in the Baltic Sea in early summer (June–July, transect and time series data), but were relatively frequent in late summer (3–30 August, time series data; Figures 5a–d). In addition, type I sequences represented a minor part of the 2009 transect data but showed a higher abundance in the mid- to late summer in the 2011 time series (Figures 5a and b).

## Discussion

### Distribution of pigment phenotypes and ecological significance

Our analyses of PC and PEI subunit phylogenies (Figures 4a and b), assembled PBS gene clusters (Figure 4c) and distribution patterns of pigment types (Figure 5) showed that a coherent clade of PEI-containing picocyanobacteria with a previously unseen PBS gene cluster organization (type IIB) dominate the low-salinity and brackish areas of the Baltic Sea. Core-genome phylogenetic analysis suggests that these picocyanobacteria belong to an uncultivated clade within subcluster 5.2 (Figure 2). In contrast to the brackish waters of the Baltic Sea, type III genotypes (containing PEII) dominated in samples from the western marine Baltic Sea area and were present in lake Torne Träsk (Figures 3 and 5). This suggests that picocyanobacteria in these environments are adapted to absorbing light in the blue spectrum, consistent with the more clear water in these ecosystems (Holmberg, 2007; Doron *et al.*, 2011), and warrants further investigation of the presence of PEII-containing picocyanobacteria in other oligotrophic fresh water environments. Furthermore, the majority of picocyanobacteria in the marine coastal waters were closely related to CA4 strains. Although the data do not allow us to determine whether these sequences in fact belong to chromatic acclimators, high rates of water mixing may have promoted the development of this picocyanobacterial phenotype, a phenomenon observed previously (Palenik, 2001).

The dominance of PEI-containing picocyanobacteria in the brackish Baltic Sea suggests that the majority of cells exhibit a red pigment phenotype. This corroborates earlier findings from the southern Baltic Sea, where up to 90% of *Synechococcus* cells were of red pigmentation (Mazur-Marzec et al., 2013), but contrasts those from the eastern Gulf of Finland where red and green picocyanobacteria were found (based on flow cytometry fluorescence) to be approximately equally abundant in the water column but with depth-driven distribution patterns (Haverkamp *et al.*, 2009). The waters in the Gulf of Finland are more turbid with a shallower euphotic zone compared with samples in our study (Fleming-Lehtinen and Laamanen, 2012), possibly shifting the light spectrum in the Gulf of Finland toward red light that favours green strains. As the light spectrum was not investigated in our study, we cannot determine its effect on the PC/PEI subunit distribution. However, we found no change in PC/PEI subunit ratio across different depths nor any depth-driven structuring of either PC or PEI phylogenies. Type IIB contigs contained both PC and PEI subunits and linker genes *cpcCD* and *cpeCE*, a gene complement not seen in any other picocyanobacterial reference genome (Figure 4c). This gene complement opens the possibility that type IIB picocyanobacteria may synthesize PE-containing PBS rods with multiple PC discs, possibly giving cells unique light absorption characteristics. All previously investigated PEI-containing *Synechococcus* lack *cpcCD* linker genes and consequently are incapable of building rods containing more than one PC disc (Six *et al.*, 2007). However, certain cyanobacteria outside the picocyanobacterial cluster 5, such as *Fremyella diplosiphon*, contain rods with multiple PC discs, providing greater capacity for red light absorption (Grossman, 2003). In addition, this organism is able to alter the PC:PEI ratio in the PBS rods via a mechanism known as type III chromatic acclimation (CA3) (Kehoe and Gutu, 2006). CA3 allows cells to respond to changes in light quality by synthesizing PBS with rods composed of PC under red light and of both PC and PEI under green light, thereby shifting the colour of cells from green to red. This represents a different form of chromatic acclimation than the CA4 response found in some cluster 5 picocyanobacteria (Palenik, 2001; Gutu and Kehoe, 2012). It is not known whether picocyanobacteria with the novel type IIB gene cluster organization discovered here are capable of altering their PBS PC:PEI ratio, but the unique presence of both PC- and PEI-associated subunits and linker genes in these genomes at least raises the possibility for such a scenario. It should be pointed out, however, that we could not identify a second set of *cpcBA* genes, critical for CA3 function in *Fremyella*, in our data set, nor homologues of *Fremyella* CA3 regulatory genes. Further analyses of type IIB picocyanobacteria, including transcriptomics and proteomics of isolates, is needed to test this hypothesis. Nevertheless, our data show that the Baltic Sea is a particularly suited environment for future analyses of the type IIB clade as these genotypes dominate and may be specifically adapted to both near- and off-shore waters in this area (Figure 5).

### Evolution of the PBS gene cluster and the role of HGT

It has been hypothesized that the ancestor of the 5.1 and 5.2 subclusters harboured PEI and that this phenotype was lost in type I subcluster 5.2 strains (Everroad and Wood, 2006). Our results, based on expanded sets of both whole-genome and metagenomic sequence data, support such a scenario. The topology of the PC phylogeny (Figure 4a) is consistent with evolution of type I PBS gene clusters from type II strains via gene loss in a common ancestor. The contrasting core-genome and PBS subunit phylogenies shown here for the subcluster 5.2 strain CB0205 (Figures 2a, 4a and b) suggests that the PEI genes were re-acquired in this strain from a marine representative. The congruent phylogenetic patterns for Baltic Sea type II picocyanobacteria and high similarity to strain CB0205 further suggests that such PEI gene re-acquisition occurred before the diversification of type II picocyanobacteria in the Baltic Sea. The question is then whether the type IIB genotypes of the Baltic Sea lost and then re-acquired their PEI genes or the PEI genes were kept from the common ancestor of the cluster 5. Inspection of the organization of the type IIB gene cluster provides multiple lines of evidence supporting HGT. First, the gene organization upstream of the *cpcGII* gene at the start of the type IIB gene cluster was identical to other subcluster 5.2 genomes but contained a repeated gene segment that included the *tRNA-Phe* gene (Figure 4c). tRNA genes frequently act as integration sites for genetic elements, which may transfer additional genes between donor and recipient (Williams, 2002). Second, the %GC of the region upstream of *cpcGII* differed from the rest of the PBS gene region (64% and 56%, respectively). Third, two transposases, common mobile genetic elements, were found between the conserved hypothetical *unk5* and the *cpeC* linker. Finally, two *cpcGII* genes with conflicting phylogenies were identified at either end of the type IIB gene cluster (Figure 4c; Supplementary Figure S4B). Gene transfer between bacteria is often mediated by phage infection and several PBS genes have been found in cyanophage infecting picocyanobacterial strains (Mann *et al.*, 2005; Sullivan *et al.*, 2005; Clokie and Mann, 2006; Weigele *et al.*, 2007). We could not detect any PBS genes within the viral population (<0.1 μm tangential flow filtration concentrate), nor any prophage sequences within PBS contigs in either the 2009 or 2011 assemblies, suggesting that potential phage-mediated gene transfers have not been fixed within the Baltic Sea picocyanobacterial population. The position of contigs containing both PC and PEI subunits within either the type II or type IIB clade was congruent between the respective phylogenies (Figures 4a and b), which argues against recent transfer of either subunit pair between the clades. It is thus unclear from where the type IIB operon was acquired. Everroad and Wood (2012) proposed that the AS *Cyanobium* lineage acquired PEI from marine subcluster 5.1 strains, a possibility that cannot be excluded for the type IIB genotypes. However, the placement of the AS *Cyanobium* strains in the PEI phylogeny (Figure 4b) and results from analyses of alternative PEI phylogenies (Supplementary Figure S3) suggest that the type IIB gene cluster resulted from genetic exchange with members of a marine/brackish *Cyanobium* lineage. Importantly, the AS *Cyanobium* strains were well separated from Baltic Sea picocyanobacteria in a RpoC1 phylogeny (Supplementary Figure S2), supporting HGT rather than evolution from a common ancestor of PBS genes in PEI-containing *Cyanobium* (including the Baltic Sea type IIB picocyanobacteria). Notably, PC and PEI subunit-encoding reads from the low-salinity sample GS666 (2.9 PSU) in the Bothnian Bay failed to assemble with the other major contigs in the transect data, indicating that these sequences belong to a distinct PEI-containing population possibly derived from riverine input (connecting limnic water bodies in the north of Sweden to the Baltic Sea). The clustering of these sequences within the type II PC clade (Figure 4a) and within the type IIB PEI clade (Figure 4b) raises the possibility that picocyanobacteria originating from freshwater sources act as genetic donors to a Baltic Sea picocyanobacterial population.

## Conclusions

PE-containing picocyanobacteria belonging to an uncultivated clade of subcluster 5.2 and harbouring a novel PBS gene cluster constituted a reoccurring and dominant portion of the picocyanobacterial population in the brackish Baltic Sea. The unique presence of both PC and PE rod linker genes suggests that members of this clade may utilize PBS with rods composed of PE and multiple PC discs, possibly giving cells new light-harvesting capabilities. Furthermore, we conclude that PC-containing subcluster 5.2 picocyanobacteria evolved from a PE-containing ancestor via loss of PE-associated genes and that multiple HGT events have re-introduced parts of the PBS gene cluster into coastal brackish picocyanobacteria.

## Figures and Tables

**Table 1:** Characteristics and gene content of the different pigment types

	*Type I*	*Type II*	*Type III (a–d)*
Appearance colour	Blue-green	Red	Orange-red
Light absorption colour	Red	Yellow-green	Blue-green
Absorption maximum	600–660 nm	570 nm	495, 550 nm
PBS Phycobiliprotein	PC (*cpcBA*)	PC (*cpcBA*) PEI (*cpeBA*)	PC (*cpcBA*) PEI (*cpeBA*) PEII (m*peBA*)
PBS rod main chromophore	PCB	PEB	PEB/PUB
PBS rod linkers	*cpcCD*	*cpeCE, mpeD*^a^	*cpeCE, mpeCDE*^b^*, mpeFG*^c^

Abbreviations: PC, phycocyanin; PCB, phycocyanobilin; PE, phycoerythrin; PEB, phycoerythrobilin; PUB, phycourobilin.

a Lacks PUB-binding domain.

b *mpeC* is not present in the type III strain WH7803.

c *mpeFG* is only present in some type III strains.

**Table 2:** Picocyanobacterial reference genomes used for comparisons

*Name*	*Abbreviation*	*Subcluster*	*Pigment type*	*Isolation source*	*Reference/institute*
*Synechococcus* sp. WH 7803	WH7803	5.1	IIIa	Sargasso Sea	Dufresne *et al.* (2008)
*Synechococcus* sp. CC9605	CC9605	5.1	IIIc	California Current	Dufresne *et al.* (2008)
*Synechococcus* sp. WH 8102	WH8102	5.1	IIIc	Tropical Atlantic	Palenik *et al.* (2003)
*Synechococcus* sp. CC9311	CC9311	5.1	IIId	California Current	Palenik *et al.* (2006)
*Synechococcus* sp. CC9902	CC9902	5.1	IIId	California Current	Dufresne *et al.* (2008)
*Synechococcus* sp. RS9916	RS9916	5.1	IIId	Red Sea	Dufresne *et al.* (2008)
*Synechococcus* sp. BL107	BL107	5.1	IIId	Blanes Bay, Mediterranean Sea	Dufresne *et al.* (2008)
*Synechococcus* sp. WH 7805	WH7805	5.1	II	Sargasso Sea	Dufresne *et al.* (2008)
*Synechococcus* sp. RS9917	RS9917	5.1	I	Red Sea	Dufresne *et al.* (2008)
*Synechococcus* sp. WH 8016	WH8016	5.1		Woods Hole	JGI
*Synechococcus* sp. WH 8109	WH8109	5.1		Sargasso Sea	JCVI
*Synechococcus* sp. CC9616	CC9616	5.1		California Current	JGI
*Synechococcus* sp. RCC307	RCC307	5.3	IIIb	Mediterranean Sea	Dufresne *et al.* (2008)
*Synechococcus* sp. CB0205	CB0205	5.2		Chesapeake Bay	JCVI
*Synechococcus* sp. CB0101^a^	CB0101	5.2		Chesapeake Bay	JCVI
*Synechococcus* sp. WH 5701	WH5701	5.2	I	Long Island Sound	Dufresne *et al.* (2008)
*Cyanobium gracile* PCC 6307	PCC6307	5.2	I	Freshwater	Shih *et al.* (2013)
*Cyanobium* sp. PCC 7001	PCC7001	5.2	I	Long Island Sound	JCVI

Abbreviations: JCVI, J Craig Venter Institute, USA; JGI, Joint Genome Institute, USA; PBS, phycobilisomes.

a Not used for PBS analysis.
