# Supplementary figures and images for: Picocyanobacteria containing a novel pigment gene cluster dominate the brackish water Baltic Sea
Source: ISME J. 2014 Mar 13;8(9):1892–903. doi: 10.1038/ismej.2014.35 (PMC4139726; doi:10.1038/ismej.2014.35)

# cpcBA-IGS %GC

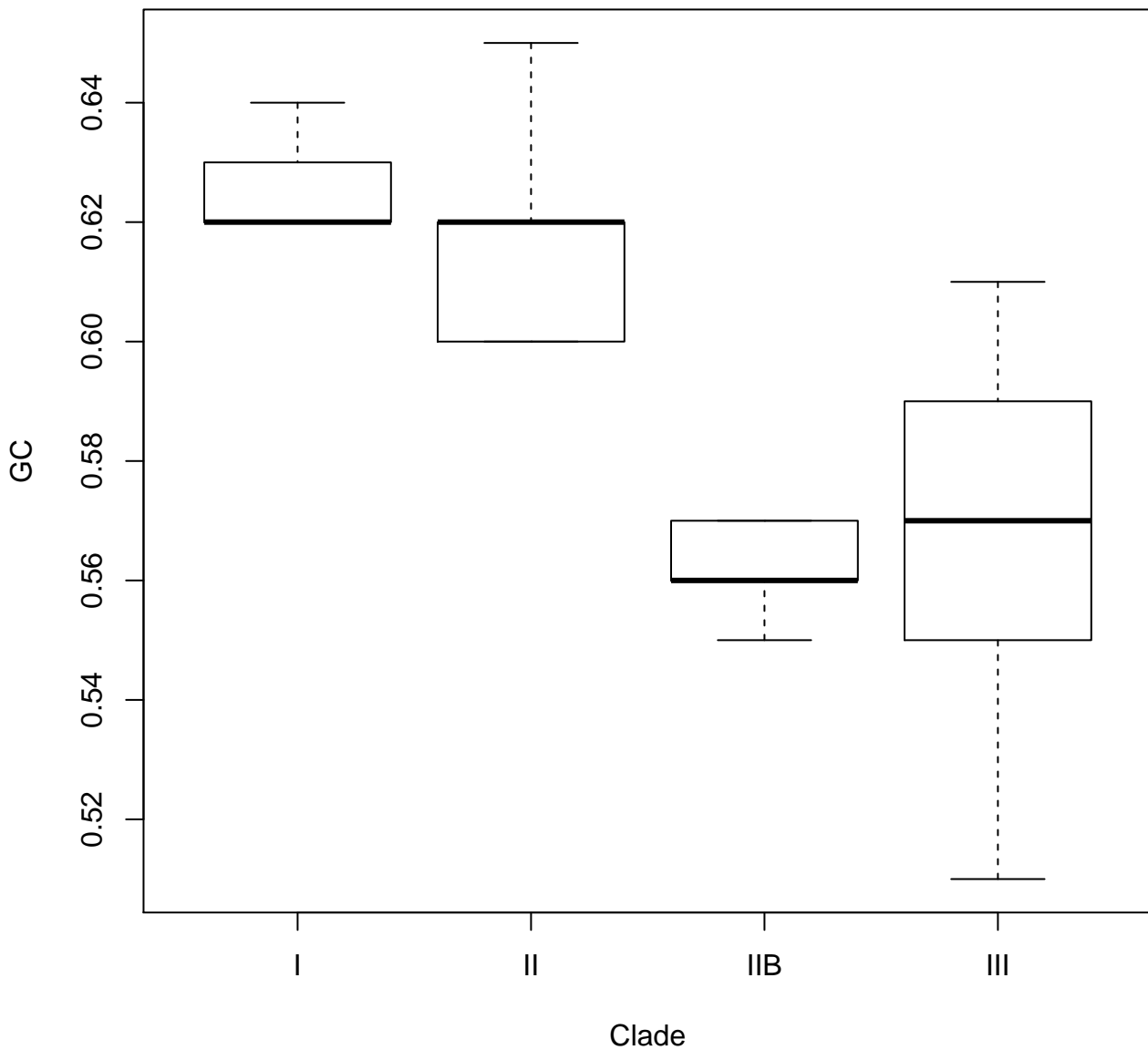

Supplement: Supplementary Figure 1 [file ismej201435x1.pdf]

# RpoC1

## RNA polymerase subunit

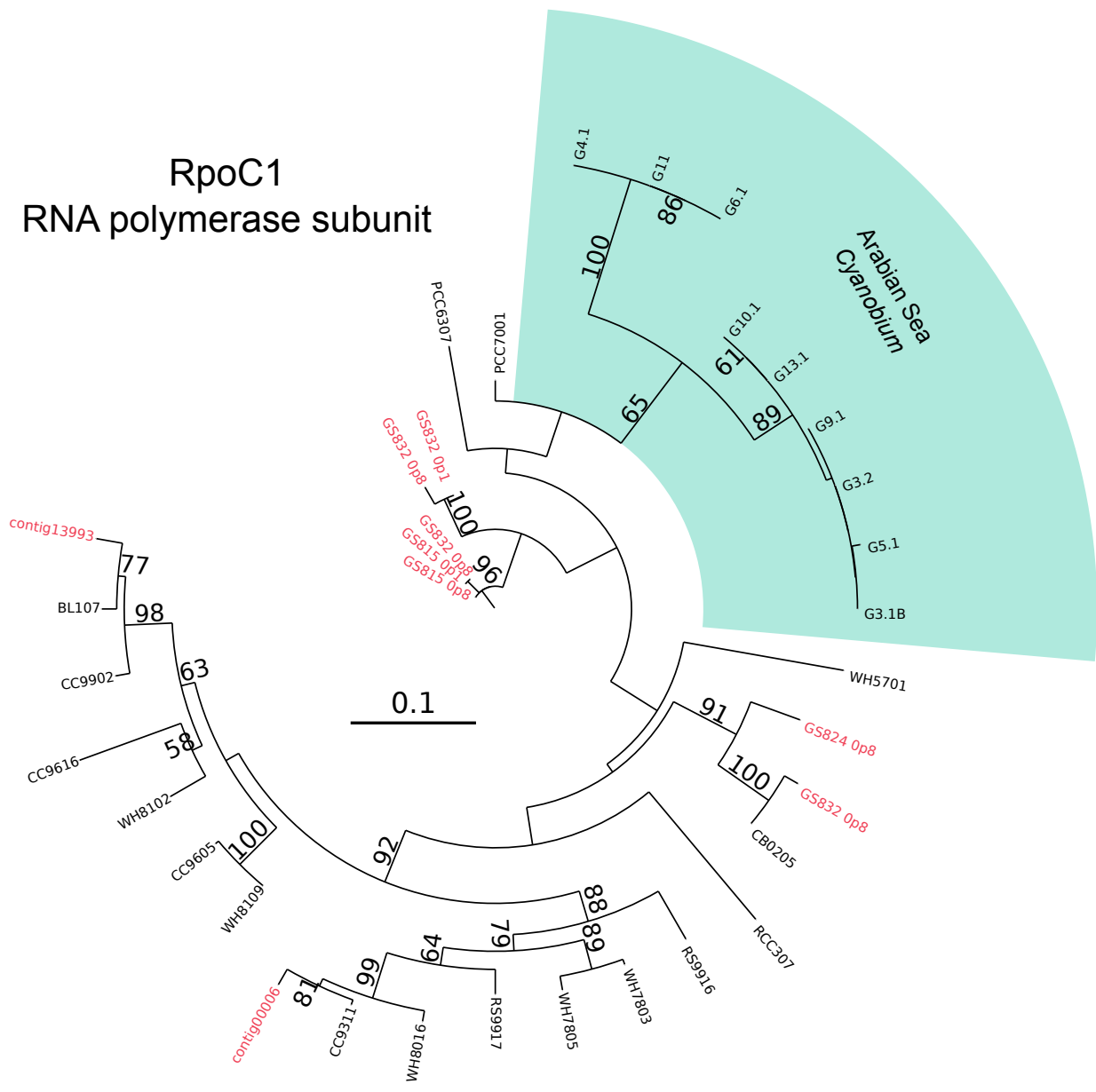

Supplement: Supplementary Figure 2 [file ismej201435x2.pdf]

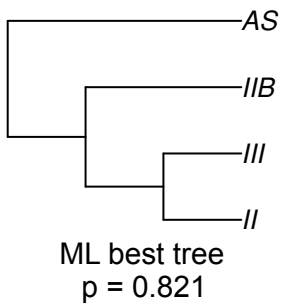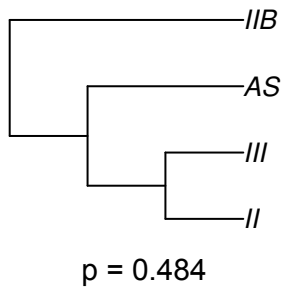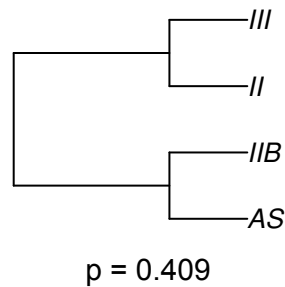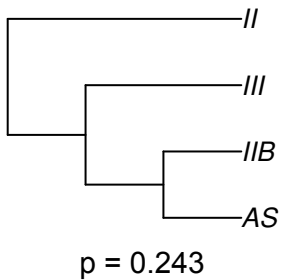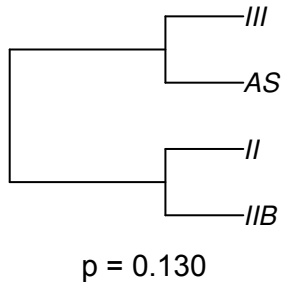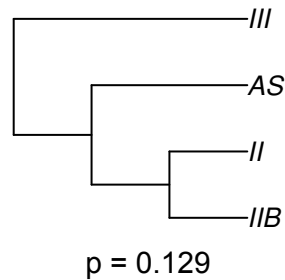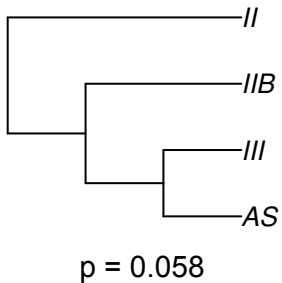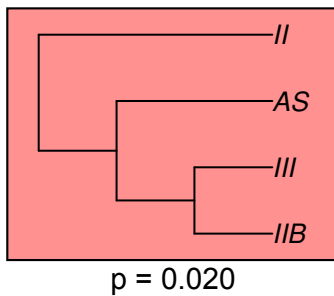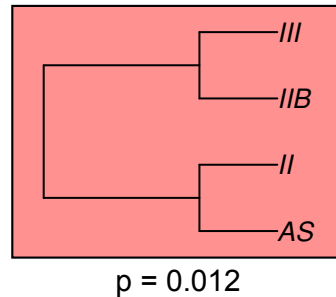

Supplement: Supplementary Figure 3 [file ismej201435x3.pdf]

**A**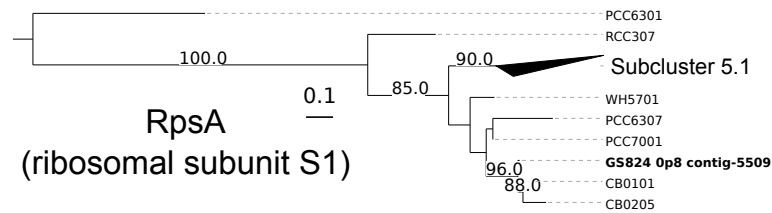**B**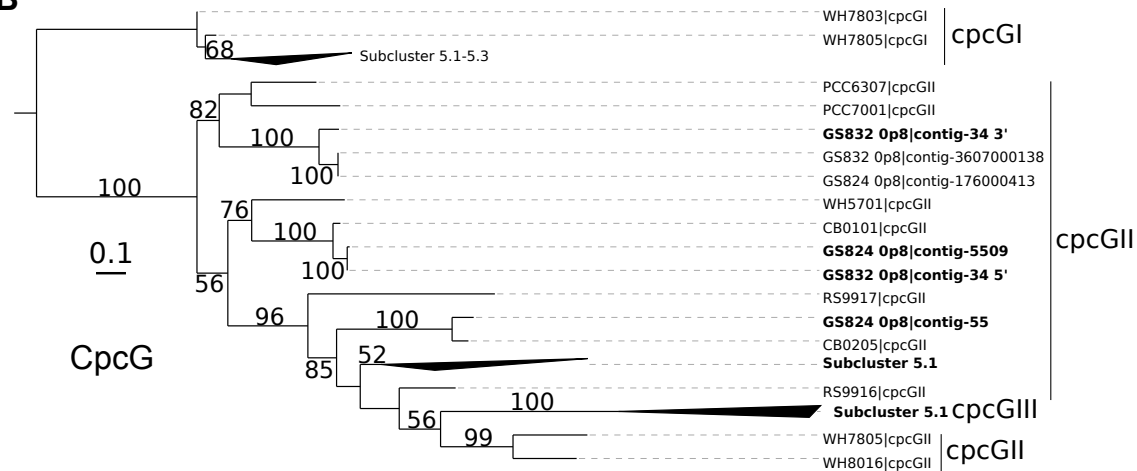**C**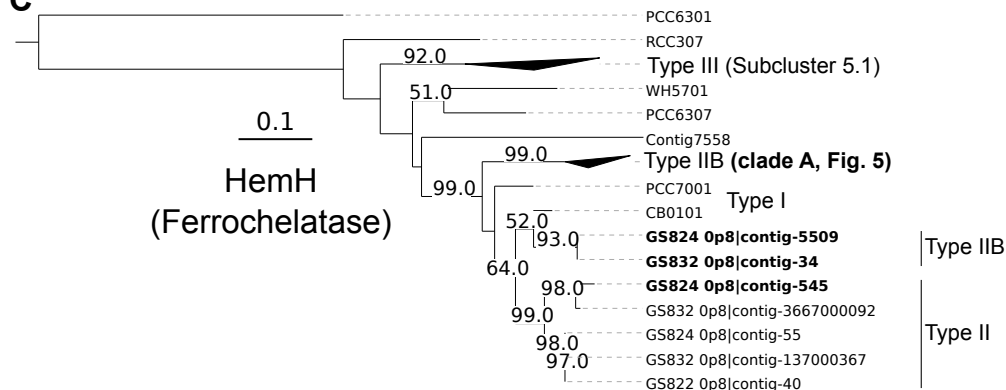**D**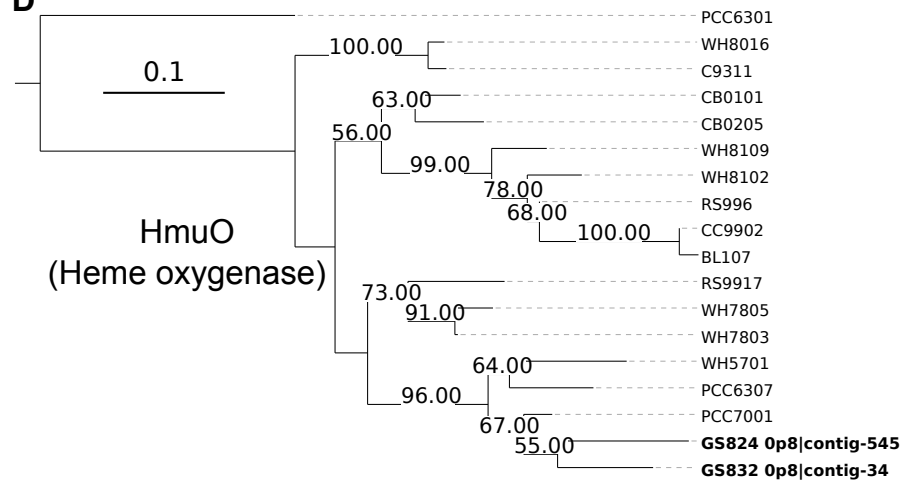

Supplement: Supplementary Figure 4 [file ismej201435x4.pdf]

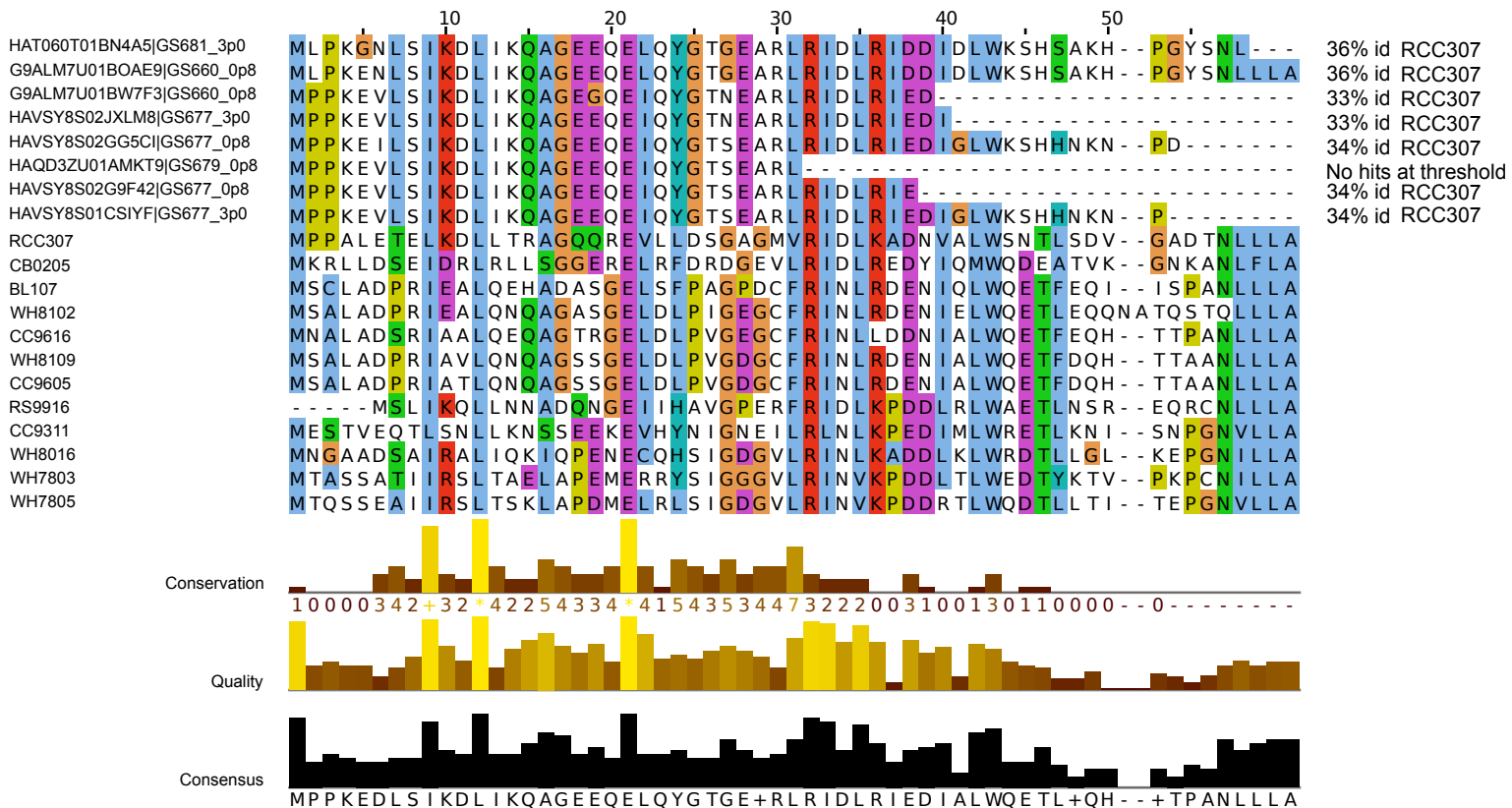

Supplement: Supplementary Figure 5 [file ismej201435x5.pdf]
