# Supplementary material for: Picocyanobacteria containing a novel pigment gene cluster dominate the brackish water Baltic Sea
Source: ISME J. 2014 Mar 13;8(9):1892–903. doi: 10.1038/ismej.2014.35 (PMC4139726; doi:10.1038/ismej.2014.35)
Supplement: Supplementary Information [file ismej201435x9.doc]

# Supplementary experimental procedures

## Sampling and sequencing

Samples for the 2011 dataset were taken from the surface layer outside the Askö marine research laboratory (coordinates 58.82 N, 17.63 E) between 09:00 AM and noon at six dates (Table S1). The samples were treated and DNA extracted as in (Rusch et al. 2007).

## Sequencing, assembly and annotation

All reads from the 2009 transect were assembled using the Newbler assembler (Margulies et al. 2005) at 98% identity threshold. A subset of 698,865 sequences were selected from the 2009 dataset based on the following criteria: 1) the read was present on a contig from the initial Newbler assembly that had a cyanobacterial tetranucleotide signature, or 2) a translated open reading frame on the read was classified as being cyanobacterial by an automated phylogenetic inference system (APIS) (Zeigler Allen et al. 2012). The cyanobacterial reads were then assembled with Newbler at 98% identity threshold and a minimum contig length of 1 kbp. This cyano-specific assembly generated 9,630 contigs. A BLASTN search with all PBS-genes from the sequenced reference genomes and the initial assembly were used to identify 37 contigs containing at least one of the PBS genes (E-value cut-off <1e-5). Nucleotide sequences for the 37 contigs were then used in a BLASTX search against the NCBI RefSeq database (downloaded on January 25, 2013 and updated with sequences from the *Synechococcus* sp. CC9616 genome obtained from the IMG database on December 3, 2012) to find open reading frames after which contigs were manually curated.

The 2011 time-series dataset contained sequences from both MiSeq and HiSeq sequencing runs of for each filter fraction in each sample. In total, this dataset comprised 1,470,228,756 read pairs. The Ray Meta assembler (Boisvert et al. 2012) was used to create separate assemblies for each filter fraction sequence set. Ray was run with 31 and 41 kmer lengths and assemblies with longest N50 lengths were chosen for each sequence set. Open reading frames were identified on contigs using FragGeneScan v. 1.16 (Rho et al. 2010). All contigs with at least one identified PBS gene were manually curated in UGENE v. 1.11.4 (Okonechnikov et al. 2012).

## PBS gene identification

PBS operon genes were identified using reciprocal best blast (RBB) searches. In a first step, protein sequences for genes found in the PBS operon of picocyanobacterial reference genomes (see Table 1) were used as queries in a BLASTP search against all metagenomic proteins with e-value cutoff <1e-10. Retained metagenomic sequences were then used to query the NCBI RefSeq database (see above). Queries with RBB hits to either of the original phycobilisome proteins were kept. For the 2009 dataset, nucleotide reads for each of the RBB hits were manually annotated in order to find reads that contained both alpha and beta subunits (for PC, PEI and PEII). For the 2011 assembly, contigs containing at least one PBS gene were manually curated.

PBS subunit sequences from previously published datasets (Table S3) were also queried in a BLASTP search against the NCBI RefSeq database as in the second step of the RBB search above. Sequences that did not have either of the picocyanobacterial reference sequences as their best hit were removed from the analysis.

## Phylogenetic analyses

The AMPHORA2 pipeline (Wu and Scott, 2012) was used to identify protein sequences from 31 conserved genes in the metagenomic datasets and in 126 cyanobacterial genomes. Metagenomic sequences classified as Cyanobacterial by AMPHORA2 were selected from this search. Six ribosomal genes (*rplEFNP* and *rpsEM*) present in all 126 cyanobacterial genomes and on 11 contigs from the 2011 assemblies were chosen from this search. Amino acid alignments were constructed using Muscle v3.8.31 (Edgar 2004). Amino acid substitution models for each alignment were selected by the Akaike Information Criterion (AIC) obtained using ProtTest v.3.0 (Darriba et al. 2011). Alignments were concatenated and a maximum likelihood (ML) phylogeny was constructed using RAxML v. 7.3.5 (Stamatakis 2006) in a partitioned setup with the corresponding amino acid substitution models, GAMMA model of rate heterogeneity and 1000 bootstrap replicates. The tree was rooted using *Gloeobacter violaceus* PCC7421.

Phycocyanin (*cpcBA*, PC) and phycoerythrin (*cpeBA*, PEI) subunits in picocyanobacterial reference genomes, and on contigs in the 2009 cyano-specific and 2011 time-series assemblies that contained both the alpha and beta subunits in each category were used to construct phylogenetic reference trees. Amino acid sequences for subunits were aligned individually with Muscle (Edgar 2004) and the alignments curated manually in Jalview (Waterhouse et al., 2009) before concatenating. Amino acid substitution model selection, RAxML analysis and rooting was performed as for the core-genome phylogeny.

Sequences for other proteins discussed in the main text were identified with RBB searches as for the PC, PEI and PEII subunits and phylogenetic analyses were performed as described above but with 100 instead of 1,000 bootstrap replicates. All trees were visualized using iTOL (Letunic & Bork 2007).

### Evolutionary placement of reads

Metagenomic protein fragments identified by AMPHORA2 as belonging to the six ribosomal proteins were aligned to the corresponding reference alignment using AMPHORA2 with default trimming. The aligned fragments were then added to the concatenated reference alignment with gaps introduced for missing data. The PC and PEI protein fragments were aligned using Muscle, manually curated and added to the corresponding reference alignment. Sequences were then added to the reference phylogeny using the EPA algorithm in RAxML (Berger et al. 2011) with all branches evaluated using slow insertions. Branches longer than two times the median branch length of all sequences in each tree were trimmed prior to calculating sample distributions.

### AU tests

Alternative PEI phylogenies were manually created in Archaeopteryx v. 0.968 (Han & Zmasek 2009) and analysed using the approximately unbiased (AU) criterion in CONSEL v. 0.20 (Shimodaira & Hasegawa 2001). Per site log likelihoods for all trees were computed using RAxML.

# Supporting figure legends

**Figure S1. Differences in %GC for clades in the PC phylogeny.** Boxplots show the %GC of major clades in the PC phylogeny (Fig. 4A), based on all included *cpcBA* operons. The %GC in the Type IIB clade operons was significantly lower than in the Type I and Type II clades (Wilcoxon rank sum test, p<0.05).

**Figure S2. RpoC1 phylogeny.** Maximum-Likelihood tree based on aligned amino acid sequences for the RNA polymerase RpoC1 subunit. Reference strain designations as in Table 1. Sequences from the 2011 time-series assemblies begin with 'GS'. The *Cyanobuim* strains from the Arabian Sea (Everroad & Wood 2006) form a monophyletic clade indicated in the figure.

**Figure S3. Alternative PE tree topologies used for AU tests.** Topologies for the PE phylogeny tested using the approximately unbiased (AU) test, ordered by decreasing likelihood scores. The best phylogeny (given in Fig. 5) is shown in thee top-left corner. Rejected phylogenies (at p=0.05) are indicated by shaded red boxes.

**Figure S4. Phylogenetic patterns of genes associated with the PBS gene cluster.** ML phylogenies based on aligned amino acid sequences for the ribosomal protein RpsA (A), the PBS core linker protein CpcGII (B), the ferrochelatase protein HemH (C) and the heme oxygenase protein HmuO (D). Bootstrap support (100 replicates) > 50% is shown at nodes. In B) the different copies of the CpcGII protein on contig 34 are indicated with 5' (start of the PBS gene cluster) and 3' (end of the gene cluster).

**Figure S5. Alignment of the translated amino acid sequence of conserved hypothetical gene *unk4*.** Shown is a protein alignment of the *unk4* product from reads also harbouring the *cpcA* gene as well as the *Synechococcus* reference sequences. Best blast hits and % identity is shown in the right margin.

# References

Berger SA, Krompass D, Stamatakis A. (2011). Performance, Accuracy, and Web Server for Evolutionary Placement of Short Sequence Reads under Maximum Likelihood. Syst. Biol. 60:291–302.

Boisvert S, Raymond F, Godzaridis É, Laviolette F, Corbeil J. (2012). Ray Meta: scalable de novo metagenome assembly and profiling. Genome Biol. 13:R122.

Darriba D, Taboada GL, Doallo R, Posada D. (2011). ProtTest 3: fast selection of best-fit models of protein evolution. Bioinforma. Oxf. Engl. 27:1164–1165.

Edgar RC. (2004). MUSCLE: multiple sequence alignment with high accuracy and high throughput. Nucleic Acids Res 32:1792–1797.

Everroad RC, Wood AM. (2006). Comparative Molecular Evolution of Newly Discovered Picocyanobacterial Strains Reveals a Phylogenetically Informative Variable Region of β-phycoerythrin1. J. Phycol. 42:1300–1311.

Han M, Zmasek C. (2009). phyloXML: XML for evolutionary biology and comparative genomics. BMC Bioinformatics 10:356.

Letunic I, Bork P. (2007). Interactive Tree Of Life (iTOL): an online tool for phylogenetic tree display and annotation. Bioinforma. Oxf. Engl. 23:127–128.

Margulies M, Egholm M, Altman WE, Attiya S, Bader JS, Bemben LA, et al. (2005). Genome sequencing in microfabricated high-density picolitre reactors. Nature 437:376–380.

Okonechnikov K, Golosova O, Fursov M. (2012). Unipro UGENE: a unified bioinformatics toolkit. Bioinformatics 28:1166–1167.

Rho M, Tang H, Ye Y. (2010). FragGeneScan: predicting genes in short and error-prone reads. Nucleic Acids Res. http://nar.oxfordjournals.org/content/early/2010/08/29/nar.gkq747 (Accessed August 8, 2013).

Rusch DB, Halpern AL, Sutton G, Heidelberg KB, Williamson S, Yooseph S, et al. (2007). The Sorcerer II Global Ocean Sampling Expedition: Northwest Atlantic through Eastern Tropical Pacific. PLoS Biol 5:e77.

Shimodaira H, Hasegawa M. (2001). CONSEL: for assessing the confidence of phylogenetic tree selection. Bioinforma. Oxf. Engl. 17:1246–1247.

Stamatakis A. (2006). RAxML-VI-HPC: maximum likelihood-based phylogenetic analyses with thousands of taxa and mixed models. Bioinformatics 22:2688–2690.
